# Theory of mind in schizophrenia through a clinical liability approach: a sib-pair study

**DOI:** 10.3389/fpsyg.2024.1391646

**Published:** 2024-12-13

**Authors:** M. Giralt-López, S. Miret, S. Campanera, M. Moreira, A. Sotero-Moreno, MO. Krebs, L. Fañanás, M. Fatjó-Vilas

**Affiliations:** ^1^Servei de Psiquiatria Infantil i de l’Adolescència, Hospital Universitari Germans Trias i Pujol, Badalona, Spain; ^2^Departament de Psiquiatria i Medicina Legal, Universitat Autònoma de Barcelona, Bellaterra, Barcelona, Spain; ^3^Institut Recerca Germans Trias i Pujol (IGTP), Badalona, Spain; ^4^Centre de Salut Mental d’Adults de Lleida, Servei de Psiquiatria, Salut Mental i Addiccions, Hospital Universitari Santa Maria, Lleida, Spain; ^5^Centro de Investigación Biomédica en Red de Salud Mental (CIBERSAM), Instituto de Salud Carlos III, Madrid, Spain; ^6^Institut de Recerca Biomèdica (IRB) de Lleida, Lleida, Spain; ^7^FIDMAG Germanes Hospitalàries Research Foundation, Barcelona, Spain; ^8^Université Paris Cité, Institute of Psychiatry and Neuroscience of Paris (INSERM U1266), GHU-Paris Psychiatrie et Neurosciences, Paris, France; ^9^Departament de Biologia Evolutiva, Ecologia i Ciències Ambientals, Facultat de Biologia, Universitat de Barcelona, Barcelona, Spain

**Keywords:** schizophrenia, theory of mind, basic symptoms, psychotic-like experiences, unaffected siblings, endophenotype

## Abstract

**Background:**

Consistent findings indicate that Theory of Mind (ToM) is impaired in schizophrenia (SZ). To investigate whether such deficits are trait- or state-dependent, we investigated if ToM is modified by clinical liability markers (such as basic symptoms and psychotic-like experiences), focusing on the analysis of unaffected siblings of individuals diagnosed with SZ.

**Methods:**

The study included a total of 65 participants: 38 patients diagnosed with a schizophrenia-spectrum disorder and 27 healthy siblings. ToM was assessed using the Hinting Task (HT), Basic symptoms with The Frankfurt Complaint Questionnaire (FCQ), Psychotic-like-experiences with the Community Assessment of Psychic Experiences (CAPE) and Family history with the Family Interview for Genetic Studies.

**Results:**

First, a comparison of HT performance between patients and siblings (linear mixed model adjusted for age, sex and Intelligence Quotient (IQ)) showed that patients presented lower scores than siblings (*p* = 0.022). These differences did not remain significant after adjusting for clinical vulnerability markers. Second, within siblings, linear regression analyses (adjusted for age, sex, IQ and family history) showed that higher FCQ Depressiveness and CAPE negative scores were related to poorer ToM performance (*p* = 0.007 and *p* = 0.032, respectively).

**Conclusion:**

Our findings suggest that clinical liability markers are valuable for delineating variations in ToM capabilities within healthy individuals. Moreover, our results indicate that ToM deficits are not solely linked to SZ but also extend to its clinical vulnerability, suggesting that ToM could serve as an endophenotypic marker. This implies that ToM could help distinguish particularly susceptible individuals from a population at risk, such as those with a genetic predisposition (siblings).

## 1 Introduction

Social cognition encompasses a diverse range of skills that facilitate the perception, interpretation and processing of social stimuli and enable individuals to interact with one another and function in society (Frith and Frith, 2007). Growing evidence indicates that schizophrenia (SZ) is associated with impaired social cognition, emerging as a predictor of the disorder outcome, even more than neurocognition (Fett et al., 2011; Lewandowski et al., 2020).

The neurodevelopmental model of SZ posits that the illness results from deviations of neurodevelopmental processes that commence years before symptom onset due to a complex interplay of numerous susceptibility genes and environmental factors (Birnbaum and Weinberger, 2017). While the cerebral organization of social processes remains incompletely understood, the maturation of social cognition during childhood and adolescence suggests that proper brain development may be pivotal in its acquisition and performance. These considerations jointly point towards the presence of neurodevelopmental disturbances that could influence both the risk of developing a psychotic disorder and the social cognition deficits associated with it. In this regard, some structures and neural networks implicated in SZ are also crucial for the development of social cognition skills, such as the prefrontal cortex (Weinberger, 2002; Teffer and Semendeferi, 2012; Green et al., 2015) or the default mode network (Salgado-Pineda et al., 2011; Schurz et al., 2014; Soares et al., 2023), which adds evidence to the potential overlap between the disorder and the neurobiological substrate of the social brain.

Theory of Mind (ToM) is one of the various domains of social cognition, including the ability to deduce the mental states of others, such as their beliefs, intentions, desires, and emotions (Frith and Frith, 1999). ToM impairments are reported in SZ (Brune, 2005; Bora and Pantelis, 2013; Chung et al., 2014; Mondragón-Maya et al., 2017; Giralt-López et al., 2020). Some investigations interpreted such deficits as a state marker (linked to symptomatology) (Mazza et al., 2012; Balogh et al., 2014). Conversely, other research characterizes ToM impairments as a trait marker, pointing to data indicating that it is not modulated by clinical severity or insight (Sprong et al., 2007; Ay et al., 2016; Giralt-López et al., 2020), or to the stability observed after a longitudinal three-year follow-up design (Ayesa-Arriola et al., 2014).

In line with the view as a trait marker, several studies also indicate that ToM deficits are present in first-episode psychosis (FEP) and high-risk individuals (unmedicated prodromal subjects) (Bora and Pantelis, 2013; Healey et al., 2016). Based on these data, the aforementioned deficits in ToM have been proposed as a potential endophenotype for SZ-spectrum disorders. On this subject, an essential aspect to investigate the properties of ToM as an intermediate phenotype is to examine the performance of healthy relatives compared to both patients and control groups. Meta-analytical findings on social cognition among unaffected relatives of individuals with SZ have revealed their poorer performance compared to that of healthy controls (Lavoie et al., 2013), and that the level of impairment in relatives lies somewhere between the levels seen in patients and controls (Bora and Pantelis, 2013). However, other more recent studies show heterogeneity, and some studies fail to demonstrate this intermediate position of the relatives’ group (Fett and Maat, 2013; Cassetta and Goghari, 2014; Ho et al., 2015; Ay et al., 2016; Raju et al., 2019; Giralt-López et al., 2020; Abreu-Fernández et al., 2023). A helpful strategy to explain the heterogeneous results in the relatives’ group and detect healthy relatives with a potentially higher load for SZ is to narrow the cohort-related confounders between patients and relatives by focusing on the sib-pairs. This way, different studies consistently report that different facets of social cognition performance in the sibling group are significantly lower than those of the control group (Ho et al., 2015; Fusar-Poli et al., 2022; Altuntaş et al., 2023).

Another strategy is to study the association between ToM and other known vulnerability markers in SZ. In this respect, we previously showed that high levels of positive and negative schizotypy are related to poorer ToM performance in relatives but not in controls (Giralt-López et al., 2020), suggesting the association of schizotypal traits with an increased risk of ToM deficits. However, previous research aiming to link social cognitive abilities with markers of familial and clinical risk for SZ, such as schizotypy and high-risk populations, has yielded inconclusive findings. These inconsistencies could be attributed to variations in the specific dimensions of social cognition assessed, as well as the diverse tasks utilized to measure the multifaceted construct of ToM. Specifically, evidence shows an association between the positive dimension of schizotypy and deficits in ToM as assessed by the Hinting Task (HT) (Gooding and Pflum, 2011). Nonetheless, no such relationship has been observed between any dimension of schizotypy and ToM when using alternative tests such as the Reading the Mind in the Eyes Test or the ToM Picture Stories Task (Gooding and Pflum, 2011; Bedwell et al., 2014; Kong et al., 2021). Moreover, at-risk populations, especially those at clinical high risk, show altered, both increased and decreased, functional activation in a range of cortical and subcortical regions during social cognition tasks, while results in familial risk populations have not driven direct conclusions (Kozhuharova et al., 2020).

Following a fully dimensional model, beyond schizotypy, other clinical markers of vulnerability have been described for SZ. For example, Basic Symptoms (BS) and Psychotic-Like Experiences (PLEs) constitute the earliest subsyndromal symptoms or cognitive impairments experienced by the patient and they are considered the most immediate symptomatic expression of the neurobiological substrate of the disease (Huber and Gross, 1989). They can be assessed concretely and consistently, providing a precise meaning of what the subject is experiencing subjectively.

The BS consist of subtle, subclinical complaints principally of volition, affect, thinking and language (speech), (body)perception, memory, motor action, central vegetative functions, control of automatic cognitive processes, and stress tolerance and represent the earliest symptoms that the patient experiences subjectively and can appear a long time before the outbreak of SZ (Huber and Gross, 1989). In this regard, a meta-analysis showed the role of BS as a risk predictor by concluding that the mean risk of transition to psychosis established from BS criteria is 48.5% (Fusar-Poli et al., 2012). Moreover, an increasing gradient of some BS was observed from non-clinical to SZ-spectrum individuals, with unaffected siblings in the intermediate position (Maggini and Raballo, 2004). Also, the offspring of parents with mood and psychotic disorders had significantly higher BS scores than control offspring (Zwicker et al., 2019), placing BS as a marker of familial risk of psychopathology. However, to our knowledge, no relationship between BS and social cognition has been established except for an association with emotion recognition processing speed in high-risk individuals (Glenthøj et al., 2020).

PLEs are subtle, subclinical hallucinations and delusions, which present in the general population with a prevalence around 6% (Linscott and van Os, 2013; McGrath et al., 2015). An extensive study reported a shared genetic liability between psychotic experiences and several psychiatric disorders (Legge et al., 2019) and another has related their presence to an increase in the risk for transition to psychotic and (to a lesser degree) non-psychotic disorder at an annual rate of 0.6% (Kaymaz et al., 2012). Some studies have shown that non-affected siblings of patients with SZ present higher PLEs scores than controls (Johnstone et al., 2000; Fekih-Romdhane et al., 2020), but another study found negative results (Landin-Romero et al., 2016). To our knowledge, only two studies have investigated the association between social cognition and the presence of PLEs in the general population, showing its association with poorer Facial Emotion Recognition and ToM (Barragan et al., 2011; Roddy et al., 2012).

Another complementary approach pursuing the understanding of the association of ToM with vulnerability to psychosis is to examine whether ToM is influenced by the genetic burden of the disease. In this sense, family history of SZ can be considered a proxy for the genetic liability background. First, because research has consistently shown that individuals with a relative with SZ have a higher risk of developing the disorder themselves compared to the general population and that it is related to the degree of relatedness (i.e., the amount of genetic variability shared with the affected relative/s) (Gottesman, 1991; Lo et al., 2020). Molecular studies have provided convergent results by reporting that first-degree relatives of patients with psychotic disorder have higher levels of polygenic risk scores (PRS) for SZ, and showing that such genetic-based risk is associated with subthreshold psychosis phenotypes (Lin et al., 2023). Therefore, while PRS of SZ mediates the family history of SZ/psychoses (Agerbo et al., 2015), it is not a necessary or sufficient factor for developing the disorder (Mars et al., 2022), due to its multifactorial and complex patterns of inheritance.

### Aims of the study

We aimed to expand our comprehension of ToM variability and its potential role as a trait marker by focusing on the unaffected siblings of individuals diagnosed with psychosis. The use of a sib-pair design is particularly relevant due to its recognized power to control for cohort and familial characteristics (Begg and Parides, 2003; Susser et al., 2010), and because healthy siblings are genetically related to affected individuals but are not exposed to potential confounders such as the pharmacological treatment (Lange et al., 2008; Glahn et al., 2019).

First, we examined differences in ToM between patients and their healthy siblings, while also exploring whether these differences persisted after considering clinical and familial risk markers for psychosis. Such clinical risk was assessed through BS and PLEs, which have proven to be effective indicators for identifying healthy individuals at risk of mental health issues compared to the broader general population (Andreou et al., 2023). Second, we explored if the ToM performance of siblings could be better understood by considering these clinical vulnerability markers and the genetic load based on the family history of psychotic disorders.

## 2 Materials and methods

### 2.1 Sample

The sample included 65 participants: 38 patients diagnosed with SZ-spectrum disorder (SSD) and 27 healthy siblings from these patients. All participants were of European origin. The sample was recruited at the Outpatient Mental Health Clinic of the Hospital Santa Maria de Lleida and assessed by the same clinician (SM). This belongs to a family-based sample already described in Giralt-López et al. (2020). In this study, with the intention of reducing the heterogeneity of the group of healthy relatives and some confounding factors (such as age or others related to cohort-effects), we have focused on studying new risk markers in the group of unaffected siblings.

The patients’ siblings underwent a clinical interview, and those without current or lifetime psychotic spectrum or affective disorder diagnoses were included in the study.

Both siblings and patients fulfilled the following exclusion criteria: (i) intellectual disability or any major medical illness that could affect brain function, (ii) neurological conditions and history of head trauma with loss of consciousness.

### 2.2 Assessments

The patients were diagnosed according to DSM-IV-TR criteria and interviewed using the Comprehensive Assessment of Symptoms and History (CASH) (Andreasen et al., 1992). It consists of almost 1000 items, most of them with detailed clinical definitions, which are scored with a Likert-type scale with values from 0 to 5. The CASH facilitates the possibility of adopting a dimensional perspective (mania, depression, catatonia, disorganization, psychosis and negative dimensions).

Symptom severity and prevalence of positive or negative symptoms were assessed by means of The Positive and Negative Syndrome Scale (PANSS) (Peralta Martín and Cuesta Zorita, 1994). It is a 30-item scale designed to measure positive, negative and general psychopathology symptoms in patients with SZ. The scores for these scales are arrived at by summation of ratings across component items. Also, a Composite Scale is scored by subtracting the negative score from the positive score. This yields a bipolar index, which is essentially a difference score reflecting the degree of predominance of one syndrome (positive or negative) in relation to the other.

Siblings were evaluated through an interview following the Structured Clinical Interview for DSM Disorders (SCID) (First and Gibbon, 2004), with the aim of ruling out the presence of a psychotic spectrum disorder. SCID is a structured interview guide for making diagnoses according to the diagnostic criteria published in the Diagnostic and Statistical Manual for Mental Disorders (DSM) of the American Psychiatric Association.

Social cognition, specifically ToM, was assessed through the Spanish version of the Hinting Task (HT) (Corcoran et al., 1995; Gil et al., 2012), a test that consists of 10 brief stories involving two people in a conversation. The task consists of inferring what a person is implying indirectly. In each item, a correct answer gives two points (for a total of 20 points). In case of an incorrect answer, an additional hint is given, after which a correct answer gives one point. The task has good validity and has proven sensitive to ToM difficulties in a number of studies to date (Corcoran and Frith, 2003).

BS were assessed by means of the Spanish version of the Frankfurt Complaint Questionnaire (FCQ), named Inventario Psicopatológico de Frankfurt, by Peralta and Cuesta (2003). The FCQ contains 98 statements describing particular complaints, each rated from 0 to 4 (never, sometimes, usually, always), indicating either the presence or absence and frequency of the complaint.

For the analyses, the structure of factors was used. In accordance with the original validation, a solution of 4 main dimensional factors was obtained (Süllwold, 1986). F1 Central cognitive disorders (11 items), F2 Perception and motor skills (15 items), F3 Depressiveness (14 items), F4 Internal and external overstimulation (9 items). This four-factorial model has provided the best fit for the 98-items version, in both first-episode of psychosis and at-risk mental state populations. The psychometric properties of these factors have been reviewed by other authors (Cuesta et al., 1996; Jimeno Bulnes et al., 1996).

Also, most of the factorial aggrupations of the FCQ have demonstrated good or excellent reliability (Uttinger et al., 2018). Interestingly, FCQ has also shown a moderate-strong long-term test–retest reliability (Loas et al., 2011).

PLEs were assessed by means of the Community Assessment of Psychic Experiences (CAPE) (Stefanis et al., 2002). This self-report questionnaire measures the lifetime prevalence of PLEs on a frequency scale ranging from ‘never’ to ‘nearly always’. The positive dimension of the CAPE includes items mainly referring to subclinical expressions of positive psychotic symptoms such as hallucinations and delusions. Similarly, the negative dimension of CAPE includes items assessing subclinical expressions of negative psychotic symptoms such as alogia, avolition, anhedonia and lack of interest in social relationships. The instrument provides a total continuous score per dimension ranging from 20 to 80 in the positive dimension and 14 to 56 in the negative dimension. Self-reported dimensions of psychotic experiences assessed by means of the CAPE have shown to be stable, reliable and valid (Konings et al., 2006). Cronbach’s alpha reported for CAPE has a meta-analytic mean of 0.91 (SD = 0.05), while alpha values of CAPE positive and negative factors (*n* = 9) had a mean of 0.84 (SD = 0.1) and 0.81 (SD = 0.10) respectively (Mark and Toulopoulou, 2016). Furthermore, this instrument has been validated in the Spanish population (Ros-Morente et al., 2011).

Nor FCQ nor CAPE contain any item assessing the social cognition domain; thus, there are no intersections.

The Intelligence Quotient (IQ) of all participants was estimated using the Block Design and Vocabulary or Information WAIS-III subtests (Wechsler, 1997).

Family history was assessed with the Family Interview for Genetic Studies (FIGS). Following broad SZ spectrum criteria (Kendler et al., 1995), families were classified by history with binary coding, as having a positive family history when patients had at least one first- or second-degree relative with SZ, affective or non-affective psychosis, or schizotypal or paranoid personality disorder. Thus, family history values are shared by all family members.

### 2.3 Statistical analyses

All data were processed using IBM SPSS Statistics Version 25.0 (SPSS IBM, Armonk, NY: IBM Corp).

Sociodemographic and clinical data were compared between groups using Student’s *t*-test for independent samples or chi-squared tests when appropriate.

HT performance differences between patients and siblings were assessed by means of linear mixed models (LMM), with total HT as the dependent variable, family member (patients / sibling) as the fixed-effect factor, sex, age and IQ as fixed-effect covariates and family as a random effect factor (subjects nested within families). In the next step of the same model, clinical vulnerability markers (BS and PLEs) were added as fixed-effect covariates.

Within patients, the relationship between HT performance and clinical characteristics (PANSS and CASH) was tested with linear regressions (adjusted for age and sex).

Within siblings, the association of HT performance with BS and PLEs was tested with a stepwise linear regression model, including in the first step age, sex and IQ; adding in the second step the family history, and finally adding each of the clinical vulnerability factors.

## 3 Results

### 3.1 Sample characteristics

Patients were assessed when clinically stable and their DSM-IV-TR diagnoses were: schizophrenia (*n* = 32) and psychotic disorder not otherwise specified (*n* = 6). Patients’ mean age at onset was 22.12 (SD = 3.83), and the mean duration of illness was 26.40 months (SD = 24.44). All were treated with antipsychotic monotherapy: 94.7%, with second-generation antipsychotic (24 risperidone, 5 olanzapine, 3 amisulpride, 2 ziprasidone, 2 clozapine) and 5.3% with haloperidol.

None of the siblings presented a psychotic spectrum disorder, which was ruled out by means of a structured clinical interview.

The sample’s sociodemographic and clinical features are presented in Tables 1, 2. Compared to the patients, the siblings had a larger percentage of women and were older but did not show differences in years of education and IQ. As per the PANSS Composite Scale, negative symptoms were more prevalent than positive symptoms in all patients. No significant associations were found between ToM performance and PANSS or CASH scores (data not shown).

**Table 1:** Sample description and statistical comparisons between patients and siblings.

	Patients (*n* = 38)	Siblings (*n* = 27)	t/ χ^2^ (*p*-value)
Male (%)	73.68	40.7	7.14 (0.008)
Age at interview	24.92 (3.90)	28.78 (5.12)	−3.45(0.001)
Years of education	13.43 (3.12)	14.74 (4.07)	n.s.
IQ	90.03 (15.23)	93.19 (15.58)	n.s
PANSS positive	10.63 (3.36)	–	
PANSS negative	20.37 (4.01)	–	
PANSS general	32.58 (7.32)	–	
CASH mania dimension	0.79 (0.27)	–	
CASH depression dimension	1.45 (0.76)	–	
CASH catatonia dimension	0.26 (0.50)	–	
CASH psychosis dimension	0.41 (0.64)	–	
CASH disorganization dimension	0.50 (0.76)	–	
CASH negative dimension	2.01 (0.67)	–	

Proportion (%) or mean scores (standard deviation). IQ Intelligence quotient, PANSS Positive and Negative Syndrome Scale, CASH Comprehensive Assessment of Symptoms and History (Present State).

**Table 2:** Theory of Mind (HT: Hinting Task), Basic Symptoms (FCQ: The Frankfurt Complaint Questionnaire) and Psychotic-Like Experiences scores (CAPE: Community Assessment of Psychic Experiences) in patients and healthy siblings.

	Patients	Healthy siblings	t (*p*-value)
Hinting Task^a^	15.74 (4.07)	18.37 (1.47)	−4.01 (<0.001)
FCQ Central cognitive disorders^b^	9.22 (14.62)	0.83 (1.27)	3.47 (0.001)
FCQ Perception and motor skills^b^	5.84 (10.76)	0.58 (0.78)	2.96 (0.005)
FCQ Depressiveness^b^	14.19 (16.22)	2.67 (2.31)	4.23 (<0.001)
FCQ Internal and external overstimulation^b^	8.19 (9.48)	1.25 (1.48)	4.37 (<0.001)
CAPE positive^b^	25.70 (9.43)	20.71 (1.60)	3.15 (0.003)
CAPE negative^b^	25.11 (7.19)	20.63 (3.46)	3.25 (0.002)

a Available for 38 patients, 27 siblings.

b Available for 37 patients, 24 siblings.

Mean scores (standard deviation).

### 3.2 Differences in ToM performance between individuals with psychosis and healthy siblings

A comparison of HT performance between patients and siblings (adjusted by age, sex and IQ), showed that patients presented significantly lower scores than siblings (*F* = 5.56, *p* = 0.022; estimated mean difference = −2.20). None of the covariables showed a significant effect (Supplementary Table S1).

After adjusting the previous model for the clinical vulnerability markers (BS and PLEs) the differences in HT performance between patients and siblings became not significant, indicating that the initially observed HT differences between them were attributable to the clinical vulnerability (Supplementary Tables S2–S7).

### 3.3 Association between clinical liability markers and hinting task performance in healthy siblings

#### 3.3.1 Association of basic symptoms with hinting task performance

First, sex, age and IQ showed non-significant effects on HT performance (assessed with a linear regression model).

As a second step, when the family history (a proxy of the genetic load) was added to the model, it showed a significant effect (*ß* = −0.584, *p* = 0.003, R_adj_^2^ = 34.8%). This indicates an inverse relationship between genetic load and ToM abilities. In this model, age and IQ become significant (*p* < 0.05), but sex did not (*p* = 0.08).

As a third step, we included the FCQ Factors (Table 3). Higher scores on the Depressiveness factor (F3) were associated with worse HT performance (*ß* = −0.444, *p* = 0.007). This model explains 54.4% of the HT variance (R_adj_^2^), which represents a significant increase in the explained variance as compared with the previous model (ΔR^2^ = 18.2%).

**Table 3:** Results of the linear regression analysis of family history and FCQ Depressiveness factor (The Frankfurt Complaint Questionnaire, F3) on Hinting Task performance in healthy siblings.

	Standardized ß coefficient	*p*-value
Age at interview	−0.438	0.011
Sex	0.444	0.011
IQ	0.498	0.005
Family history	−0.616	0.001
FCQ depressiveness	−0.444	0.007

IQ Intellectual quotient.

No relationship was observed with the other FCQ factors (Supplementary Tables S8–S10).

#### 3.3.2 Association of psychotic-like experiences with hinting task performance

Following the same approach as in the previous section, we assessed the role of the family history and the clinical liability measured with the CAPE (Table 4). We observed that higher scores on the CAPE Negative dimension were associated with worse HT performance (*ß* = −0.357, *p* = 0.032). This model explains 47% of the HT variance (R_adj_^2^), which represents a significant increase in the explained variance as compared with the previous model (ΔR^2^ = 12.4%). No relationship was observed with CAPE positive scores (Supplementary Table S11).

**Table 4:** Results of the linear regression analysis of family history and Community Assessment of Psychic Experiences (CAPE) negative dimension on Hinting Task performance in healthy siblings.

	Standardized ß coefficient	*p*-value
Age at interview	−0.395	0.028
Sex	0.325	0.064
IQ	0.422	0.020
Family history	−0.623	0.001
CAPE negative	−0.357	0.032

IQ Intellectual quotient.

## 4 Discussion

This study aimed to improve the understanding of the ToM variability by investigating whether ToM is influenced by genetic and clinical liability markers of SZ in healthy relatives (siblings).

Previous research findings regarding social cognition in unaffected relatives of individuals with SZ have yielded inconclusive results, potentially attributed to several factors such as varying sample sizes, diverse tasks used for ToM assessment, and the characteristics of the comparison group comprising relatives. While most studies have examined first-degree relatives, limited research has specifically targeted the siblings. Therefore, the current study, based on a sib-pair approach, enriches the findings regarding the differences detected in ToM between patients and relatives reported in our previous study (Giralt-López et al., 2020), thanks to: (i) controlling for relevant cohort-effects related to ToM competence such as age and educational level (Moran et al., 2012; Cassidy et al., 2020; Velthorst et al., 2023), (ii) refining the characterization of the ToM heterogeneity by assessing the role of both clinical liability and genetic load markers on the HT performance.

When we analysed whether HT performance differed between unaffected and affected siblings, we first observed that the healthy ones presented higher scores than patients. This is in line with previous meta-analytic data, reporting that the social cognition abilities in relatives lie somewhere between the levels seen in patients and healthy non-relatives (Bora and Pantelis, 2013; Lavoie et al., 2013); although these results are not consistently replicated (Fett and Maat, 2013; Cassetta and Goghari, 2014; Ho et al., 2015; Ay et al., 2016; Raju et al., 2019; Giralt-López et al., 2020). Second, when we assessed the role of clinical liability in such a between-groups effect, we observed that HT performance differences between patients and siblings became not significant. Hence, we interpret that, when accounting for clinical markers (here, BS and PLEs), the priorly observed differences are blurred, probably reflecting a similar performance between patients and a subgroup of siblings especially vulnerable to the disease.

To interpret this finding, we have considered other possible factors, such as the potential effect of medication. In this sense, it is necessary to highlight that the evidence seems to indicate that antipsychotic medication would improve ToM (Javed and Charles, 2018; Kimoto et al., 2018), possibly by stabilizing symptoms. Therefore, while the non-inclusion of the treatment data in our analyses could limit the interpretation, we could hypothesize that if considered the patients’ performance on ToM could be potentially poorer, potentially revealing greater disparities with the siblings. Also, other factors such as educational level, age and environment could influence the evaluation of ToM. In this sense, the sib-pair design is the best fitted to control for this type of cohort or stratification confounders since siblings are more likely to have grown up in a similar environment, have a smaller age difference (compared to parent–child designs), and are more likely to have received a similar educational style. Nevertheless, the effect of age and IQ has been considered in the models. Conversely, we did not include the educational level because of its association with IQ in our sample (increasing the risk of collinearity) and the non-different mean years of education between our groups (Table 1).

Focusing on the healthy siblings, our data showed that familial and clinical liability markers for SZ were related to ToM performance. In the first instance, the analysis of age, sex and IQ variables did not show an effect on healthy siblings HT scores. Next, when the family history was included in the model, we observed that a higher familial load was significantly related to lower ToM scores, which highlights the relationship between genetic vulnerability in the sibling group and poorer ToM skills.

Interestingly, we also report that the addition to the models of the FCQ Depressiveness Factor and the CAPE negative scores improved the explained variance of ToM, with the higher scores in both factors being significantly related to lower HT performance. Thus, our data seems to point towards the role of SZ genetic and clinical proneness in modulating the ToM in healthy individuals.

While the limited sample size of the study warrants cautiousness in the interpretation of our findings, it is remarkable that they are consistent with not only our previous study (Giralt-López et al., 2020), but also with other studies describing the association of other clinical liability markers for SZ with social cognition deficits in relatives (Irani et al., 2006) and the general population (Bora, 2020). In all, these findings add evidence of the interest in considering different vulnerability markers to characterize the underlying heterogeneity of ToM. Also, they indicate the interest of further analyses to elucidate the role of this heterogeneity as a potential explanation of the divergent results across studies evaluating social cognition within healthy or at-risk relatives. In this direction, longitudinal studies of siblings might disentangle the pattern of ToM in those who would develop or not psychosis.

Regarding the specific association of the dimensions reflecting subclinical expressions of negative symptoms (FCQ Depressiveness and CAPE negative dimension), it is partially consistent with accumulated evidence suggesting that ToM deficits are linked to negative (and disorganization) symptoms (Urbach et al., 2013; Ventura et al., 2013; Mehta et al., 2014; Bliksted et al., 2017; Yolland et al., 2020). The same specificity has been described in social cognition dimensions other than ToM, such as emotion perception or management (Charernboon and Patumanond, 2017; Yolland et al., 2020).

Moreover, the fact that all patients in our sample were in remission state and showed more prevalent negative than positive symptoms, and that ToM performance was not modulated by clinical severity (PANSS and CASH), seems to support that ToM behaves as a trait variable associated with the disease or its vulnerability more than a state variable associated with acute episodes. Nevertheless, a few studies have previously described significant associations between positive symptoms and ToM performance (Sprong et al., 2007; Mehl et al., 2010; Fretland et al., 2015; Bliksted et al., 2017). One study reported the association of poorer ToM performance with positive symptoms due to a specific error type (overmentalising instead of reduced ToM) (Fretland et al., 2015). Bliksted et al. (2017) noted that only in the presence of high levels of negative symptoms, positive symptoms were associated with deficits in social cognition. These findings lend evidence to a more complex pattern of ToM performance in SZ than only a matter of impaired versus non-impaired ToM.

As regards the characterization of ToM or other domains of social cognition concerning the genetic load for SZ (family history), as already mentioned before, some molecular-based approaches have contributed to defining the relationship between the genetic liability for SZ and social cognition (Lin et al., 2023). Estimating the individual genetic risk for SZ through the Polygenic Risk Score (PRS-SZ) has provided information on the link between the genetic risk for the disorder and the development of aspects of social cognition such as emotion identification speed (Germine et al., 2016). Also, the PRS-SZ has been associated with differences in the interregional correlations (assessed through both structural and functional neuroimaging approaches) between the core and other face-processing brain regions in healthy young adults (Lieslehto et al., 2019), and inversely associated with emotional recognition in healthy subjects but not in patients affected by SZ (Tripoli et al., 2022).

From the perspective of the genetic load assessed through familial relatedness and high-risk conditions, Tikka et al. (2020) conducted a study comparing ToM performance among patients with psychosis, a group of individuals at clinical high risk for psychosis, a cohort of siblings, and a control group. Their findings indicated that patients and both at-risk groups (familial and clinical) exhibited poorer ToM performance compared to controls, with no significant differences observed between the patient group and either of the risk groups (Tikka et al., 2020). Building upon this framework, our study contributes to the examination of ToM variability by juxtaposing patients with those at familial risk (siblings) and considering clinical liability markers.

With all the aforementioned, our data suggest that ToM deficits could be a promising indicator of those subjects within a population at risk (in this case, genetic risk due to the fact of being siblings) who have a greater risk for psychosis and on whom monitoring measures could be applied for early detection. The considerable negative impact of cognitive impairment (neurocognition and social cognition) on different domains of real-world outcomes (Fett et al., 2011; Silberstein and Harvey, 2019), and our current lack of efficient treatment strategies emphasize the importance of identifying the underlying mechanisms of cognitive phenotypes in psychotic disorders as a crucial step towards understanding the aetiology of these disorders (Owen et al., 2016).

To contextualize our findings, it is crucial to consider the strengths and limitations of the study. The strengths include the family-based design and the use of an affected-unaffected sibling approach, and the inclusion of clinical and genetic liability markers together with social cognition data. Notably, only a limited number of studies have investigated the correlation between social cognition and BS or PLEs, with none incorporating data on genetic liability (family history). Consequently, our study design and the phenotypic variability contribute to the ongoing debate regarding state versus trait characteristics and offer insights into the complexity of traits associated with SZ liability.

On the other hand, although the sample size is comparable to that of previous studies and a common limitation of family-based designs is the difficulty in recruiting large, well-characterized families, the size of our sample remains the main limitation of the study. This issue could result in an overestimation of the effect size, and, therefore, requires caution in the interpretation of the findings as well as indicates the need for larger samples. Likewise, the sample size has prevented the analyses separately by-sex, affecting the generalization of the results. The current design could also be improved by the inclusion of a control group, which would allow testing the intermediate position of relatives between patients and controls. Additionally, while ToM is a crucial aspect of social cognition, our study did not explore other domains, such as emotion processing and social knowledge.

Finally, in terms of the translation of our results, identifying ToM deficits can facilitate the development and implementation of tailored interventions for individuals with psychosis or those in at-risk mental state. As highlighted, deficits in ToM can significantly affect social functioning, thus targeted rehabilitation in this area establishes a new avenue for intervention in psychosis and for individuals at heightened risk.

Improving ToM skills can also enhance engagement with therapy and treatment programs for psychosis because of better communication and an improved ability to understand others’ viewpoints, ultimately leading to higher receptiveness to therapeutic interventions and more favorable treatment outcomes. Furthermore, the improvement in ToM abilities can empower individuals with psychosis to advocate for themselves in various social and clinical settings and to actively participate in their treatment and daily lives, thereby enhancing their overall quality of life.

## 5 Conclusion

Overall, our findings contribute to the ongoing discussion surrounding the potential for social cognition, specifically ToM, to serve as an endophenotypic marker of psychotic disorders.

In particular, our data indicate the usefulness of clinical liability markers in characterizing differences in ToM abilities within healthy individuals and suggest that deficits in ToM are not only associated with SZ-spectrum disorders but with clinical vulnerability for this disorder. These findings support that ToM could behave as an endophenotypic marker and contribute to discriminating especially vulnerable subjects from a population at risk, in this case, genetic risk (siblings). Nevertheless, new analyses in larger samples are needed.

## Data Availability

The dataset generated for this study is available on request to the corresponding authors.
