# Supplementary material for: Theory of mind in schizophrenia through a clinical liability approach: a sib-pair study
Source: Front Psychol. 2024 Dec 13;15:1391646. doi: 10.3389/fpsyg.2024.1391646 (PMC11672793; doi:10.3389/fpsyg.2024.1391646)
Supplement: Supplementary file 1 [file Table_1.docx]

**SUPPLEMENTARY MATERIAL FOR:**

**Theory of mind in schizophrenia through a clinical liability approach: a sib-pair study**

*M Giralt-López ; S Miret; S Campanera; M Moreira ; A Sotero-Moreno ; MO Krebs ; L Fañanás; M Fatjó-Vilas*

**Table S1.** Results of the linear mixed model, with total HT as the dependent variable, family status (patients / sibling) as the fixed-effect factor, sex, age and IQ as fixed-effect covariates and family as the random effect (subjects nested within families).

|  | **Estimated effect** | **F** | **p-value** |
| --- | --- | --- | --- |
| **Age at interview** | 0.0439 | 0.211 | 0.648 |
| **Sex** | 0.400 | 0.209 | 0.649 |
| **IQ** | 0.0403 | 2.104 | 0.152 |
| **status** | **-2.203** | **5.557** | **0.022** |

**Table S2.** Results of the linear mixed model, with total HT as the dependent variable, family status (patients / sibling) as the fixed-effect factor, sex, age and IQ as fixed-effect covariates, family as the random effect (subjects nested within families) adjusted for Community Assessment of Psychic Experiences (CAPE) negative dimension.

|  | **Estimated effect** | **F** | **p-value** |
| --- | --- | --- | --- |
| **Age at interview** | 0.0520 | 0.262 | 0.611 |
| **Sex** | 0.112 | 0.014 | 0.906 |
| **IQ** | 0.0513 | 3.030 | 0.087 |
| **CAPE Negative** | -2.951 | 2.439 | 0.124 |
| **status** | -1.824 | 2.763 | 0.102 |

**Table S3.** Results of the linear mixed model, with total HT as the dependent variable, family status (patients / sibling) as the fixed-effect factor, sex, age and IQ as fixed-effect covariates, family as the random effect (subjects nested within families) adjusted for Community Assessment of Psychic Experiences (CAPE) positive dimension.

|  | **Estimated effect** | **F** | **p-value** |
| --- | --- | --- | --- |
| **Age at interview** | 0.042 | 0.167 | 0.684 |
| **Sex** | 0.188 | 0.037 | 0.848 |
| **IQ** | 0.044 | 2.234 | 0.141 |
| **CAPE Positive** | -0.890 | 0.200 | 0.657 |
| **status** | -2.205 | 3.983 | 0.051 |

**Table S4.** Results of the linear mixed model, with total HT as the dependent variable, family status (patients / sibling) as the fixed-effect factor, sex, age and IQ as fixed-effect covariates, family as the random effect (subjects nested within families) adjusted for FCQ Central cognitive disorder factor (The Frankfurt Complaint Questionnaire, F1).

|  | **Estimated effect** | **F** | **p-value** |
| --- | --- | --- | --- |
| **Age at interview** | 0.0578 | 0.336 | 0.565 |
| **Sex** | 0.324 | 0.120 | 0.730 |
| **IQ** | 0.043 | 2.186 | 0.145 |
| **FCQ Central Cognitive** | -0.425 | 0.944 | 0.335 |
| **status** | -1.632 | 2.015 | 0.161 |

**Table S5.** Results of the linear mixed model, with total HT as the dependent variable, family status (patients / sibling) as the fixed-effect factor, sex, age and IQ as fixed-effect covariates, family as the random effect (subjects nested within families) adjusted for FCQ Perception and motor disorder factor (The Frankfurt Complaint Questionnaire, F2).

|  | **Estimated effect** | **F** | **p-value** |
| --- | --- | --- | --- |
| **Age at interview** | 0.047 | 0.222 | 0.639 |
| **Sex** | 0.325 | 0.121 | 0.729 |
| **IQ** | 0.046 | 2.432 | 0.125 |
| **FCQ Perception and motor** | -0.402 | 0.697 | 0.407 |
| **status** | -1.832 | 2.830 | 0.098 |

**Table S6.** Results of the linear mixed model, with total HT as the dependent variable, family status (patients / sibling) as the fixed-effect factor, sex, age and IQ as fixed-effect covariates, family as the random effect (subjects nested within families) adjusted for FCQ Depressiveness factor (The Frankfurt Complaint Questionnaire, F3)

|  | **Estimated effect** | **F** | **p-value** |
| --- | --- | --- | --- |
| **Age at interview** | 0.065 | 0.421 | 0.519 |
| **Sex** | 0.381 | 0.166 | 0.685 |
| **IQ** | 0.047 | 2.527 | 0.118 |
| **FCQ Depressiveness** | -0.430 | 0.730 | 0.397 |
| **status** | -1.578 | 1.663 | 0.203 |

**Table S7.** Results of the linear mixed model, with total HT as the dependent variable, family status (patients / sibling) as the fixed-effect factor, sex, age and IQ as fixed-effect covariates, family as the random effect (subjects nested within families) adjusted for FCQ Internal and external overstimulation factor (The Frankfurt Complaint Questionnaire, F4)

|  | **Estimated effect** | **F** | **p-value** |
| --- | --- | --- | --- |
| **Age at interview** | 0.051 | 0.259 | 0.613 |
| **Sex** | 0.360 | 0.148 | 0.702 |
| **IQ** | 0.048 | 2.543 | 0.116 |
| **FCQ Internal and external overstimulation factor** | -0.310 | 0.404 | 0.528 |
| **status** | -1.818 | 2.488 | 0.120 |

**Table S8.** Results of the stepwise linear regression analysis within siblings on Hinting Task performance, including in a first step age, sex and IQ; adding in a second step the family history, and finally FCQ Central cognitive disorder factor (The Frankfurt Complaint Questionnaire, F1).

|  | **Standardised**  **ß coefficient** | **p-value** |
| --- | --- | --- |
| **Age at interview** | -.437 | .047 |
| **Sex** | .332 | .093 |
| **IQ** | .376 | .059 |
| **Family History** | -.578 | .004 |
| **FCQ Central Cognitive** | -.102 | .610 |

**Table S9** Results of the stepwise linear regression analysis within siblings on Hinting Task performance, including in a first step age, sex and IQ; adding in a second step the family history, and finally FCQ Perception and motor skills factor (The Frankfurt Complaint Questionnaire, F2).

|  | **Standardised**  **ß coefficient** | **p-value** |
| --- | --- | --- |
| **Age at interview** | -.537 | .014 |
| **Sex** | .372 | .050 |
| **IQ** | .247 | .222 |
| **Family History** | -.512 | .008 |
| **FCQ Perception and motor** | -.347 | .128 |

**Table S10** Results of the stepwise linear regression analysis within siblings on Hinting Task performance, including in a first step age, sex and IQ; adding in a second step the family history, and finally FCQ Internal and external overstimulation factor (The Frankfurt Complaint Questionnaire, F4).

|  | **Standardised**  **ß coefficient** | **p-value** |
| --- | --- | --- |
| **Age at interview** | -.477 | .031 |
| **Sex** | .359 | .067 |
| **IQ** | .399 | .043 |
| **Family History** | -.579 | .004 |
| **FCQ Internal and external overstimulation factor** | -.180 | .352 |

**Table S11.** Results of the stepwise linear regression analysis within siblings on Hinting Task performance, including in a first step age, sex and IQ; adding in a second step the family history, and finally Community Assessment of Psychic Experiences (CAPE) positive dimension.

|  | **Standardised**  **ß coefficient** | **p-value** |
| --- | --- | --- |
| **Age at interview** | -0.445 | 0.024 |
| **Sex** | 0.415 | 0.036 |
| **IQ** | 0.378 | 0.046 |
| **Family History** | -0.489 | 0.014 |
| **CAPE Positive** | -0.278 | 0.146 |
